# Family Background Issues as Predictors of Mental Health Problems for University Students

**DOI:** 10.3390/healthcare11030316

**Published:** 2023-01-20

**Authors:** Varisara Luvira, Pat Nonjui, Nisachon Butsathon, Phahurat Deenok, Wilawan Aunruean

**Affiliations:** 1 Department of Community, Family and Occupational Medicine, Faculty of Medicine, Khon Kaen University, Khon Kaen 40002, Thailand; 2 Family Practice Department, Division of Nursing, Srinagarind Hospital, Khon Kaen 40002, Thailand; 3 123 Primary Care Unit, Division of Nursing, Srinagarind Hospital, Khon Kaen 40002, Thailand

**Keywords:** family characteristics, universities, students, mental health

## Abstract

Mental health problems are common among university students. Specific type of family background is one of the important factors contributing to these problems. This study aimed to evaluate the proportion of severe mental health problems and the associations between severity and types of problems and family backgrounds. This was a cross-sectional descriptive study. We reviewed the database and medical records of 125 university students aged over 18 years who attended the mental health consultation clinic for university students, 123 Primary Care Unit, Khon Kaen University, between 1 January and 31 December 2018. The characteristics of the participants were summarized using descriptive statistics. We performed an analysis using logistic regression to obtain the crude and adjusted odds ratio. The proportion of severe mental health problems was 50.4%. The most common problem was learning problems (54.4%). The severity of the problems reported by the students was associated with communication failure in the family (AOR = 3.30 [95% CI: 1.14–9.52], *p* = 0.027). All students who experienced domestic violence in their family had severe mental health problems. This study re-appraised the utility of the context of the family as a predictor of current problems of university students.

## 1. Introduction

University students are at an age that transitions them from being adolescents to being young adults, who not only encounter major changes in many aspects of their lives (i.e., social life, friends, study patterns), but are also not fully mature [1]. Previous studies have found that mental problems are common among college students, at about one in five to one in two [2,3,4,5], including stress, anxiety, and depressive disorders [6,7,8], leading to worsening of learning outcomes [3]. These problems cause not only short-term but also long-term impacts, including on emotional health, physical health [9], and relationships [10].

Problems that university students encounter during their university life that can lead to mental problems include friendship problems, financial problems, learning problems [11], and family problems [12]. However, only a certain group of students have been found to have mental problems. This may be because each student has a different background, especially in terms of the family aspect. Studies have shown that family stress is strongly associated with mental health problems [13]. Previous studies have stated that the type of family affects mental problems in university students. For example, a student from a family with parental migration [14,15], relationship gaps within the family [16,17], financial problems [18], communication failures within the family [19], family conflict [20], and domestic violence [16,21,22] may experience difficulties from childhood to adolescence [23]. Some students remain with their family and continue to suffer from the problems of the family. Students who have encountered family problems from childhood to the present can develop certain mental complexes which affect the risk of mental problems when experiencing difficulties in life. Studies have shown that poor family environments, such as family conflict and a lack of warmth and affection, can prevent students in the family receiving emotional support, as well as have difficulty expressing their emotions, which can cause mental health problems [24].

Khon Kaen University is the oldest university in Northeast Thailand. The university students come from various provinces, especially provinces in Northeast Thailand, which have the most significant socioeconomic problems in the country. Students may come from various types of families with various family patterns and contexts. The Family Medicine Unit, Faculty of Medicine, established a mental health consultation clinic for university students. This clinic is run by family physicians with expertise in counseling at a primary care unit within the university. Most of the students attending this clinic come with various issues which are not severe enough to need a psychiatrist’s attention but have negative impacts on their lives as university students. Treatment consists of counseling and, if needed, medication. While running the clinic, we had noticed that most of student who had chief complaint of the mental problems, usually had some family background issue.

Our counseling process is not only focused on the current problems, but also investigates the family background issues underlying problems in order to solve issues precisely and permanently. Moreover, as family physicians, we believe in early detection and proper referral of students with severe mental health problems, who require treatment other than counseling alone. The currently available studies mention only the association between family background and the presence of problems in university students at a superficial level. There have been few reports specifically about the severity and the types of problems. This study therefore aimed to evaluate the proportion of students with severe mental health problems. Moreover, we aimed to preliminarily determine the associations between family background and student issues, in order to use family background as a predictor of the severity and types of student issues, as well as to prevent more negative impacts from family background on the students. Our results can be used to develop guidelines for the psychological care of university students, as well as providing basic information to promote and prevent psychological problems of university students.

## 2. Materials and Methods

### 2.1. Study Design and Participants

This study was a cross-sectional descriptive. Inclusion criteria were based on the database and medical records of all university students who attended the mental health consultation clinic for university students, seeking mental counseling, between 1 January and 31 December 2018. Exclusion criteria were incomplete details of the consultation in the database/medical records. The study population included 125 university students.

Data were collected from the medical records of the clinic, recorded by family physicians. These records included age, gender, faculty, student year, domicile, aspect of the problems, and family background issues.

### 2.2. Operational Definitions

Current problems in this study were defined as problems that cause anxiety, mental stress, and depressive disorders among university students, including learning problems, family problems, relationship problems, friendship problems, adaptation problems, and financial problems.

Family background issues were defined as family issues existing before the students became university students. They included parental migration, relationship gaps in the family, family economic issues, betrayal in the family, communication problems in the family, domestic violence, and conflicts within the family.

The term ‘parental migration’ in this study was defined as students being raised by someone who was neither their father nor mother, because their parents went to another region for a job opportunity.

### 2.3. Dependent Variable

The factor of interest was the severity of mental health problems. Mental problems were graded as ‘mild’, which was defined as able to be handled with general counseling, and ‘severe’, which required medication treatment, perhaps in combination with cognitive behavior therapy, or needed a referral to a psychiatrist. We were also interested in the association between the severity of mental problems and the type of family background issues. We were aware of the multicollinearity among the dependent variables. For example, age of the student and student year showed multicollinearity. We chose to only use student year in our analyses.

### 2.4. Potential Confounders

Confounding factors were variables that might be associated with the severity and types of mental stress. These were gender, faculty of the students, student year, and domicile. Faculty was classified as health science-related and non-health science-related faculties. Domicile was categorized as being from Khon Kaen province (where the university is located) or the northeast region of Thailand (which is the region containing Khon Kaen province), apart from Khon Kaen, and from other regions in Thailand.

### 2.5. Statistical Analysis

#### 2.5.1. Description of Demographic Characteristics

The characteristics of the participants were summarized using descriptive statistics. Mean and standard deviations were used for continuous variables, and frequency and percentages were used for categorical variables.

#### 2.5.2. Crude and Multivariable Analysis

To determine the association between the severity and type of mental problems and family background issues, the crude odds ratios and 95% confidence intervals (95% CI) were computed by bivariate logistic regression. We performed multivariate logistic regression to obtain an adjusted odds ratio and the 95% confidence interval. On multivariate analysis, we controlled for the effects of confounding variables, which were selected using the following criteria: (i) variables which were found to have a *p*-value less than 0.25 in crude analysis and ii) variables shown from previous reports to have effects on mental problems. The model fitting procedure was backward stepwise elimination.

All analyses were performed using the Stata program version 10 (Lakeway, TX, USA). All tests were two-sided and a *p* value < 0.05 was considered statistically significant.

### 2.6. Ethical Approval

This research was approved by the Khon Kaen University Ethics Committee for Human Research based on the Declaration of Helsinki and the ICH Good Clinical Practice Guidelines (No. HE621062). Additional informed consent was obtained from all individual participants for whom identifying information is included in this article.

## 3. Results

### 3.1. Demographic Data

This study collected data from the database and medical records of 125 university students range in age from 18–25 years. Of these, 76% were female. The mean age was 20.96 years (SD 1.29). Most (84.8%) were from non-health sciences faculties. The highest proportion (36%) of the students were studying in the second year. Most were from other provinces in the Northeast region apart from Khon Kaen (63.2%).

### 3.2. Problems of the Students

Among the 125 students, 63 had severe mental health problems (50.4%). There was no association between demographic data of the students and severity of the problems (Table 1). Most of the problems were related to learning (*n* = 68; 54.4%), followed by family problems (*n* = 61; 48.8%) and friendship problems (*n* = 56; 44.8%) (Table 2).

### 3.3. Associations between Family Background Issues and the Severity of Current Problems Concerning the Students

As shown in Table 3, after adjustment for sex [2,8,11], faculty, student year [7,8], and domicile of the students [8], the severity of the problems reported by the students was associated with communication failures in the family (AOR = 3.30 (95% CI: [1.14–9.52], *p* = 0.027). All the students who experienced domestic violence in the family had severe mental health problems.

### 3.4. Associations between Family Background Issues and the Types of Current Problems Concerning the Students

As shown in Table 4, learning problems at the university were found to be unrelated to family background issues.

Family problems during the course of study of the students were significantly associated with family background issues, including relationship gaps with either their father or mother (AOR = 8.0 (95% CI: [3.2–20.8], *p* < 0.001), economic issues (AOR = 11.2 (95% CI: [2.4–53.0], *p* = 0.002), betrayal affairs (AOR = 4.5 (95% CI: [1.5–13.3], *p* = 0.007), communication failures in the family (AOR = 12.5 (95% CI: [5.1–30.4], *p* < 0.001), domestic violence (AOR = 14.7 (95% CI: [1.6–134.6], *p* = 0.017), and conflict in the family (AOR = 7.3 (95% CI: [3.2–16.8], *p* < 0.001).

Relationship problems of university students were significantly associated with a betrayal affair in the family (AOR = 2.8 (95% CI: [1.0–7.5], *p* = 0.042).

Friendship problems were significantly associated with economic issues (AOR = 3.4 (95% CI: [1.1–10.3], *p* = 0.031) and communication failures in the family (AOR = 3.2 (95% CI: [1.5–7.0], *p* = 0.004).

Problems in adapting to university life were significantly associated with relationship gaps with either the father or mother (AOR = 7.0 (95% CI: [1.5–32.4], *p* = 0.014), communication failures in the family (AOR = 4.5 (95% CI: [1.4–14.3], *p* = 0.010), and conflict in the family (AOR = 4.2 (95% CI: [1.4–12.4], *p* = 0.009).

Financial problems of students were significantly associated with parental migration (AOR = 9.8 (95% CI: [2.2–43.9], *p* = 0.003), economic issues (AOR = was 569.5 (95% CI: [21.3–15,263.0], *p* < 0.001), and conflict in the family (AOR = 6.4 (95% CI: [1.2–33.9], *p* = 0.024).

## 4. Discussion

This study collected data from the medical records of university students of Khon Kaen University, who attended the mental health consultation clinic, 123 Primary Care Unit, in 2018. This clinic is run by family physicians of the same institute who share the same approach to the family issues of university students. Moreover, these family physicians also developed the data record form of this study for completion during data collection.

We found that half of the students who attended to our clinic required specific treatment, such as medications or a referral to psychiatrist, other than counseling by their family physician alone. This was a smaller proportion than found by the National Survey of Counseling Center Directors in 2010 in the United States [25]. This may be due to this study concerning Khon Kaen University students, as students can receive counseling and treatment services at the mental health consultation clinic free of charge, thus allowing them to access treatment more quickly. When presenting earlier following onset, many mental disorders do not yet need to be treated with medication. Communication failures and domestic violence in the family had the greatest effect on severity of problems. The students who came from such families tended to lack support from their family [19,21]. A poor family environment can be a trigger for mental health problems [24]. Moreover, since the family indirectly models communication skills, a student who experienced good communication within the family will tend to have better coping skills compared with those from families who experienced communication failures.

The most common problems among the participants were learning problems, followed by family problems and friendship problems, in that order. Regarding the data collection process, individual students might have experienced more than one problem at a time. More than half of the students came with learning problems, such as worrying about learning outcomes, stress regarding upcoming examinations, worsening learning outcomes, lacking interest in their field of study, and doubt about their own knowledge. These findings were compatible with findings from previous studies that found associations of mental health problems with academic functioning [3]. The reason the most common problems were learning problems was that all participants were studying at the time of assessment [7]. The students sought support for learning issues alone or in combination with other issues. Often, learning issues preceded other issues or vice versa. This was consistent with a previous study that showed academic stress in adolescents consists of many factors, including family, friends, and the educational system [1,26]. We also found that nearly half of the students attending our clinic had friendship issues. This were compatible with findings from previous studies that found associations between friendship and mental health [27]. People of this age are usually focused on friendships, spending more time with friends. Moreover, most friends in university are new friends, with different backgrounds. Family issues were still the most prominent issue at this age. Students still depend on their parents and family, so the family inevitably plays an important role in student life. University students are prone to inner conflicts and troubles, leading to mental health problems [3,7].

We found that the family context was associated with the issues reported by the university students at our mental health consultation clinic as follows:

### 4.1. Parental Migration

Parental migration is the most significant problem in Northeast Thailand. We found that it was associated with the severity of problems and financial issues. This was compatible with findings from a previous study that showed parental migration was associated with more mental problems among university students [14,15]. Explanations for this include: (i) when this problem occurs, the students choose to consult with no one, because there is a generation gap between them and their grandparents [16], and (ii) the family from which the parents had to migrate to another region for occupation opportunities usually has some degree of financial issues [28].

### 4.2. Unfamiliarity with Their Parents

We found that unfamiliarity with parents was associated with the severity of problems, current family problems, and adaptation issues. A previous study reported that the severity of mental problems depended on relationships within the family [16,17,29]. Relationships with parents should be strong; if students feel a distance between them and their parents, it might indicate family problems. When these students face their own problems, their family is usually unable to give support [16]. Unstable family relationships can therefore lead to student inability to adapt. This was confirmed by a previous study that found self-esteem of college freshmen is highly correlated with their family’s performance [30] and promotes student adaptation. Lack of an appropriate counselor makes adaptation more complicated, because in the transition to life as a university student, people encounter changes in many aspects of their lives, including learning, friends, and their social life, that differ from secondary school life [1].

### 4.3. Financial Issues

This study found that financial issues were related to current family problems, friendship problems, and current financial problems. Since most university students still receive financial support from their families, a student from a family with pre-existing financial issues may have problems in the family, especially financial problems. Regarding the effects on friendships, there was a study that found that poverty was associated with psychosocial symptoms in adolescence [31], because students from families with financial problems were unable to share time, money, and lifestyles with their friends who had no financial issues. Moreover, financial problems lead to depression and stress [18] that might affect friendships.

### 4.4. Infidelity of the Parents

Infidelity of the parents was associated with current student family problems and relationship problems. This was compatible with a previous study that found there was an impact of parental infidelity among late adolescents in establishing relationships [32]. This stress affected children in the family. When these students are in romantic relationships, since they have no example of a successful couple [33], they have a tendency to have problems such as feeling insecure in the relationship. This can cause problems in their attitude towards love and their skill at solving relationship problems.

### 4.5. Communication Issues in the Family

Communication issues in the family were associated with the severity of problems, current family problems, friendship problems, and adaptation issues. This was compatible with a previous study that reported that communication skills within the family are associated with adolescent mental problems [19]. Failures of communication within the family causes problems similar to those arising from unfamiliarity with parents, because good communication skills in the family are associated with family strength [34]. The problems in communication in the family might cause problems in communication for the students themselves, because they lacked a suitable model for good interpersonal communication [32].

### 4.6. Domestic Violence

Domestic violence was associated with the current family problems of the student. One study reported that children’s exposure to intimate partner violence and child abuse is strongly related to later psychological problems [16,21,22]. The students raised in a family with domestic violence, either physical or verbal, all indicated a degree of family problems, leading to mental problems.

### 4.7. Family Conflicts

Family conflicts were associated with the severity of the problems, current family problems, adaptation problems, and financial problems. This was compatible with a report from a previous study that family conflict is strongly associated with depression [20] and negative emotions [35] among university students. Students from conflicted families are prone to keeping problems to themselves. They do not want to add their own problems to the family problems, making their problems more severe. It was also found that family financial problems are one of the factors that cause family conflict [36]. This is because money is an important factor for the well-being of family members. Having financial difficulties can cause stress in the family, leading to family conflict. Students with family conflict often have financial problems.

### 4.8. Utilities of the Predictors for Approaching the Patients

Knowledge about the relationship between family background and current problems can be applied in practice. For example, when working with a student who reports friendship problems, the physician should explore economic and communication issues, which predispose students to having additional problems that should be further explored, including financial, family, and adaptation problems. The use of this approach helps to minimize the chance of missing problems. This could be applied as a simple tool for physicians to approach the mental problems of university students, and as basic information for developing prevention policies.

### 4.9. Strengths and Limitations of This Study

This study collected data in a mental health consultation clinic at the largest university in Northeast Thailand. No expenses were accrued for any student attending the clinic. The consultants were expert family physicians, making this data representative of the general population of students. We found that family background and context could be used as predictors of the current problems of the attending student. To the best of our knowledge, there is neither this kind of study nor another equivalent service in our country, making our findings unique. However, there were some limitations to this study, including that it was retrospective in nature, which might introduce some bias. Since we collected data and undertook sample recruitment to descriptively determine only the proportion of severe mental health problems and their association with family backgrounds, the association between family background and types of problems should be interpreted carefully because of the insufficient sample size. It should be confirmed with a more prospective study focusing on each problem.

## 5. Conclusions

In conclusion, this study demonstrated that half of the students who attended our mental health consultation clinic required specific treatment, such as medication or a referral to a psychiatrist, other than counseling by a family physician alone. Family context, such as parental migration, unfamiliarity with parents, financial issues, family conflicts, communication failures in the family, infidelity of the parents, and domestic violence, were associated with mental health problems in university students. This study re-appraised the utility of contexts of the family as predictors of current mental health problems in university students.

## Figures and Tables

**Table 1:** Demographic characteristics for those who had mild versus severe mental health problems.

Characteristics	Mild (*n* = 62)	Severe (*n* = 63)	*p* Value ^1^
Gender: Male	17 (27.42)	13 (20.63)	0.375
Age (years)			
18–21	42 (67.74)	45 (71.43)	0.654
22–25	20 (32.26)	18 (28.57)	
Faculty			
Health science	11 (17.74)	8 (12.70)	0.432
Non-health science	51 (82.26)	55 (87.30)	
Student year			
1–2	32 (51.61)	32 (50.79)	0.927
3–5	30 (48.39)	31 (49.21)	
Domicile			
Khon Kaen	16 (25.81)	9 (14.29)	0.270
Northeast region (apart from Khon Kaen)	36 (58.06)	43 (68.25)	
Other regions	10 (16.13)	11 (17.46)	

^1^ Chi squared test.

**Table 2:** Demographic data according to the problems of the students.

Variable	Learning (*n* = 68)	Family (*n* = 61)	Couple Problems (*n* = 36)	Friendship Relations (*n* = 56)	Adaptation (*n* = 22)	Financial Problems (*n* = 10)
Gender: Male	17 (25%)	13 (21.3%)	11 (30.6%)	15 (26.8%)	3 (13.6%)	3 (30%)
Age (years)						
18	0 (0%)	1 (1.6%)	0 (0%)	0 (0%)	0 (0%)	0 (0%)
19	7 (10.3%)	6 (9.8%)	7 (19.4%)	9 (16.1%)	4 (18.2%)	1 (10%)
20	19 (27.9%)	13 (21.3%)	4 (11.1%)	15 (26.8%)	5 (22.7%)	4 (40%)
21	24 (35.3%)	22 (36.1%)	12 (33.3%)	15 (26.8%)	9 (40.9%)	3 (30%)
22	10 (14.7%)	10 (16.4%)	8 (22.2%)	8 (14.3%)	1 (4.55%)	2 (20%)
23	5 (7.4%)	7 (11.5%)	3 (8.3%)	6 (10.7%)	1 (4.55%)	0 (0%)
24	2 (2.9%)	2 (3.3%)	2 (5.6%)	2 (3.6%)	1 (4.55%)	0 (0%)
25	1 1 (1.5%)	0 (0%)	0 (0%)	1 (1.8%)	1 (4.55%)	0 (0%)
Faculty						
Health science	15 (22.1%)	8 (13.1%)	2 (5.6%)	9 (16.1%)	3 (13.6%)	1 (10%)
Non-health science	53 (77.9%)	53 (86.9%)	34 (94.4%)	47 (83.9%)	19 (86.4%)	9 (90%)
Student year						
1	10 (14.7%)	12 (19.7%)	4 (11.1%)	11 (19.6%)	6 (27.3%)	2 (20%)
2	27 (39.7%)	19 (31.2%)	10 (27.8%)	19 (33.9%)	8 (36.4%)	5 (50%)
3	18 (26.5%)	15 (24.6%)	12 (33.3%)	13 (23.2%)	7 (31.8%)	1 (10%)
4	12 (17.7%)	14 (22.9%)	9 (25%)	11 (19.6%)	1 (4.6%)	2 (20%)
5	1 (1.5%)	1 (1.6%)	1 (2.8%)	2 (3.6%)	0 (0%)	0 (0%)
Domicile						
Khon Kaen	9 (13.2%)	8 (13.1%)	9 (25%)	11 (19.6%)	3 (13.6%)	1 (10%)
Northeast region (apart from Khon Kaen	48 (70.6%)	43 (70.5%)	22 (61.1%)	31 (55.4%)	15 (68.2%)	9 (90%)
Other regions	11 (16.2%)	10 (16.4%)	5 (13.9%)	14 (25%)	4 (18.2%)	0 (0%)

**Table 3:** Adjusted odds ratio for severe mental health problem associations with family background issues.

Family Background Issue	Mild (*n* = 62)	Severe (*n* = 63)	Crude OR	Adjusted OR ^†^	95% CI	*p* Value
Parental migration	6	16	3.18	2.01	0.59–6.74	0.258
Relationship gap within family	29	53	6.03	1.87	0.59–5.89	0.288
Economic	5	13	2.96	1.80	0.51–6.31	0.358
Betrayal	10	13	1.35	0.59	0.19–1.77	0.345
Communications	18	45	6.11	3.30	1.14–9.52	0.027 *
Domestic violence	0	10	-	-	-	-

* Results were considered significant at *p* < 0.05. ^†^ After adjustment for sex, faculty, student year, and domicile of the students.

**Table 4:** Associations between family background issues and current problems concerning the student.

Family Background Issue	Learning Problems	Family Problems	Couple Problems	Friendship Relations Problems	Adaptation Problems	Financial Problems
	Adjusted odds ratio (95% CI) ^†^					
Parental migration	1.5 (0.6–4.1)	2.6 (0.9–7.4)	1.3 (0.5–3.7)	1.2 (0.5–3.1)	1.6 (0.5–5.4)	9.8 (2.2–43.9) *
Relationship gap within family	0.7 (0.3–1.6)	8.0 (3.2–20.8) *	1.6 (0.7–3.9)	1.8 (0.8–4.1)	7.0 (1.5–32.4) *	2.6 (0.5–13.4)
Economic	0.9 (0.3–2.7)	11.2 (2.4–53.0) *	2.8 (0.9–8.6)	3.4 (1.1–10.3) *	2.2 (0.4–7.4)	569.5 (21.3–15,263.0) *
Betrayal	0.5 (0.2–1.3)	4.5 (1.5–13.3) *	2.8 (1.0–7.6) *	0.9 (0.3–2.3)	1.1 (0.3–3.8)	3.5 (0.9–14.0)
Communications	0.9 (0.4–1.9)	12.5 (5.1–30.4) *	1.4 (0.6–3.3)	3.2 (1.5–7.0) *	4.5 (1.4–14.3) *	2.7 (0.6–11.4)
Domestic violence	1.1 (0.3–4.8)	14.7 (1.6–134.6) *	0.3 (0.0–3.0)	1.5 (0.4–6.2)	2.0 (0.4–10.0)	3.2 (0.5–19.8)
Conflict within family	1.6 (0.7–3.3)	7.3 (3.2–16.8) *	0.8 (0.4–1.9)	1.4 (0.6–2.9)	4.2 (1.4–12.4) *	6.4 (1.2–33.9) *

* Results were considered significant at *p* < 0.05. ^†^ After adjustment for sex, faculty, student year, and domicile of the students.

## Data Availability

The datasets used and/or analyzed during the current study are available from the corresponding author on reasonable request.
